# Effect of retro and forward walking on quadriceps muscle strength, pain, function, and mobility in patients with knee osteoarthritis: a protocol for a randomized controlled trial

**DOI:** 10.1186/s12891-016-1021-z

**Published:** 2016-04-12

**Authors:** Ahmad Alghadir, Shahnawaz Anwer

**Affiliations:** Rehabilitation Research Chair, Department of Rehabilitation Sciences, College of Applied Medical Sciences, King Saud University, P.O.Box-10219, Riyadh, Saudi Arabia; Dr. D. Y. Patil College of Physiotherapy, Dr. D. Y. Patil Vidyapeeth, Pune, India

**Keywords:** Osteoarthritis, Knee, Walking, Exercise

## Abstract

**Background:**

Walking, a closed kinetic chain (CKC) activity, is widely used in knee rehabilitation as it allows early weight bearing and movement. It has been suggested that retro-walking may provide additional benefits beyond those experienced by forward-walking. The present study will investigate the effect of retro- and forward-walking on quadriceps muscle strength, pain, function, balance and mobility in knee Osteoarthritis (OA) subjects.

**Methods/Design:**

Sixty-nine participants with knee OA will be recruited from the outpatient department in this randomized controlled trial. The participants will be randomly assigned to one of three groups; retro walking, forward walking or control group. The training program will be 3 days/week for 6 weeks. In addition, all the participants will receive a standard physiotherapy training program. An independent assessor blinded to group assignment will measure quadriceps muscle strength, knee pain intensity, functional disability, and mobility at baseline and 6 weeks after training.

**Discussion:**

The results of this study will enhance our understanding on the therapeutic effects of walking (retro- or forward-walking) in knee OA. The findings from this study will help determine whether retro- or forward-walking or both are effective in the rehabilitation of subjects with knee OA.

**Trial registration:**

Controlled Trials ISRCTN12850845, Registered 26 January 2015.

## Background

Knee osteoarthritis (OA) causes chronic disability in the older population worldwide [1, 2]. Its prevalence increases dramatically with age. Prevalence of radiographic OA is estimated at 80 % of all adults at or over the age of 65 years [3, 4]. The common impairments such as knee pain, decreased functional mobility, quadriceps strength, and stiffness leading to physical disability have been associated with knee OA [5–7].

In knee OA, the medial compartment is affected more often than the lateral. This may be due to higher load transfer through the medial compartment compared to the lateral, thereby producing higher knee adduction torque. A previous study suggested that the initial peak knee adduction torque during walking strongly predicted the severity and rate of progression of medial knee OA [8].

The focus of knee rehabilitation exercise has gradually shifted from open kinetic chain (OKC) to closed kinetic chain (CKC) exercises, which are more functional and could be safe and effective [9, 10]. In addition to increasing muscle strength, CKC exercise could also facilitate joint position sense [11–13]. Walking, a CKC exercise, is widely used in knee rehabilitation programs as it allows early weight bearing and mobilization. Published guidelines recommend regular walking exercises for individuals with knee OA [14]. Previous systematic review and meta-analysis reported moderate effects of walking on pain and function in people with knee OA [15]. Another study reported improved function following supervised walking and patient education with no adverse effects on OA related symptoms [16]. It has been suggested that retro-walking may provide additional benefits beyond those experienced by forward walking in healthy adult males and females [17, 18].

Retro-walking is considered a safe closed kinetic chain exercise since the compressive forces at the patello-femoral joint are reduced [19]. Retro-walking reduces quadriceps eccentric function, while the isometric and concentric quadriceps strength are preserved [20–22]. Retro-walking training programs have been found to increase quadriceps strength [22]. In addition, the cardiopulmonary demand is higher during retro- walking as compared to forward-walking [23, 24]. Therefore, these advantages make retro-walking a safe and effective component of rehabilitation programs.

Recently, Gondhalekar et al. [25] investigated the effects of retro-walking on pain and physical function in subjects with knee OA. They concluded that 3-weeks of retro-walking as an adjunct to the conventional training program significantly improved function in subjects with knee OA. Another study reported improved postural stability following long-term aerobic walking and weight training programs in subjects with knee OA [26]. In addition, Messier et al. [27] reported improved pain, disability, and performance following combined walking and weight training program in subjects with knee OA. Furthermore, Evcik et al. [28] reported improved pain, disability and quality of life following walking and home-based exercise in subjects with knee OA. In contrast, Toda [29] reported worsening of symptoms following a walking program in individuals with knee OA. However, the participants in this study were obese women with knee OA. Therefore, an increased intensity of pain in these participants may be due to the weight bearing nature of walking. The present study will investigate the effect of retro- and forward-walking in quadriceps muscle strength, pain, function, and mobility in subjects with knee OA.

### Hypotheses

- The retro-walking training will reduce pain and improve quadriceps muscle strength, function, and mobility in subjects with knee OA.
- The forward-walking training will reduce pain and improve quadriceps muscle strength, function, and mobility in subjects with knee OA.
- The effects of the retro-walking will be greater compared to forward-walking in improvements of quadriceps muscle strength, pain, function, and mobility in subjects with knee OA.

## Methods/Design

### Trial design

This trial is a three-arm assessor-blinded randomized controlled trial (RCT) comparing retro-walking to forward-walking or control. All experiments will follow the Declaration of Helsinki. The protocol follows the CONSORT guidelines for reporting of non-pharmacological interventions [30].

### Participants

Sixty-nine participants with knee OA as per the American College of Rheumatology clinical and radiographic diagnostic criteria will participate [31]. In addition, participants must be between the ages of 40–70 years, have a body mass index (BMI) ≤ 29.9, and have a Kellgren-Lawrence radiographic grade of 1 –3 [32], as determined by the physician, to exclude end-stage knee OA patients. In case of bilateral knee involvement, the most symptomatic knee (as identified by the participant) will be evaluated. The participants will be excluded if they have a history of knee surgery to either knee within past 3 months, a systemic arthritic condition, any other muscular, joint or neurological condition affecting lower limb function, received physical therapy or an intra-articular injection in the knee within the past 3 months.

### Procedure

**Fig. 1:**
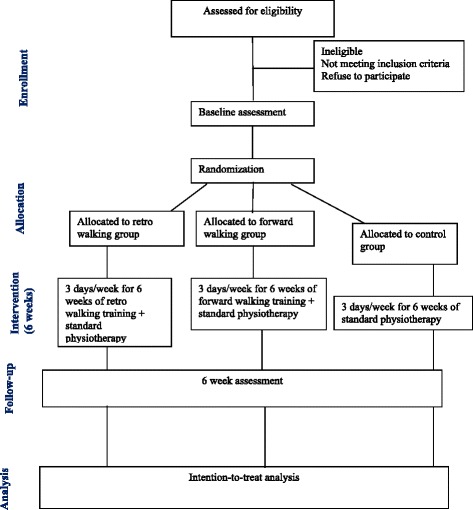
Flow of participants through each stage of the randomized trial

Figure 1 outlines the study phases. The participants who have knee OA and fulfilled inclusion criteria will participate in this study.

## Interventions

### Standard physiotherapy program

All the participants will receive a supervised standard physiotherapy training program at the outpatient physiotherapy department as reported in a previous study [33]. The exercise program will comprise a combination of OKC and CKC exercises, including isometric quadriceps, terminal knee extension, isometric hip adduction exercise, straight leg raising exercise, leg press, and semi-squat. In a recent systematic review, Anwer et al. [34] reported the use of a combination of OKC and CKC exercises in the management of subjects with knee OA. All the participants will also receive Ultrasound therapy (1.5 W/cm2 for 7 min in continuous mode) around the knee joint before exercise. A previous study suggested that the continuous mode of therapeutic ultrasound is an effective and safe treatment modality in reducing pain and improving function in subjects with knee OA [35]. The participants will perform prescribed exercises for 6 weeks (3 days/week) as previously reported [33]. All the participants will be restricted to perform a home exercise or walking program other than the prescribed program.

### Retro-walking program

The participants in group A will undergo a supervised 10 min retro-walking training with 5 min of warm-up and cool down period for 3 days a week for 6 weeks on a flat surface at their comfortable speed along with a standard physiotherapy program as mentioned above. The participants will gradually increase the walking time up to 30 min over the period of 6 weeks. In the warm up and cool down periods, the subjects will perform ankle toe movements, hamstring and gastrocnemius-soleus stretching, and heel raise exercises.

### Forward-walking program

The participants in group B will undergo a supervised 10 min of forward-walking with 5 min of warm up and cool down periods for 3 days a week for 6 weeks on a flat surface at their comfortable speed along with a standard physiotherapy program as mentioned above. The participants will gradually increase the walking time up 30 min over the period of 6 weeks. In the warm up and cool down periods, the patients will perform ankle toe movements, hamstring and gastrocnemius-soleus stretching, and heel raise exercises.

### Control group

The participants in group C will receive only a supervised standard physiotherapy program as mentioned above.

### Outcome measures

Table 1 details the outcome measures that will be assessed to determine treatment effects. The primary outcome includes knee pain intensity and functional disability. The secondary outcome includes quadriceps muscle strength and functional mobility. All measurements will be taken at baseline (week 0), and at the end of the intervention (week 6).

**Table 1:** Summary of outcome measures will be used to determine effectiveness of the treatment

Outcome	Measurement tool
Primary outcomes	
Knee pain	NRS, Reduced WOMAC pain subscale
Knee function	Reduced WOMAC physical function subscale
Secondary outcomes	
Isometric quadriceps muscle strength	Hand held dynamometer
Functional mobility	Timed up and go test

*NRS* numeral rating scale, *WOMAC* Western Ontario and McMaster Universities osteoarthritis index

### Measurement of quadriceps muscle strength

A Jamar hydraulic handheld dynamometer (Model, SH5001; SAEHAN, Changwon, South Korea) will be used to test quadriceps muscle performance. All the participants will be tested by the same physiotherapist. A test-retest reliability of therapist’s dynamometer testing will be established using 20 subjects over a period of 1 week. The participants will be instructed to sit at the edge of a treatment table with the knee in 600 of flexion using a standard goniometer. The lever arm length will be kept constant by placing the dynamometer close to the ankle at a point 80 % of the distance between the lateral malleolus and the lateral joint line of the knee. Each participant’s pelvis will be stabilized by a belt around the edge of the treatment table [36, 37].

The participants will perform four warm up contractions and will be asked to increase their knee extension force gradually over 3 s. The participants will be instructed to produce 50 % effort in the first three warm-ups and a maximal contraction on the fourth warm-up. After that, the participants will perform four maximal trials, and the average of the four trials will be used for the analysis.

### Measurement of pain intensity

Pain intensity will be assessed using a numerical rating scale (NRS) [38]. The participants will indicate their pain intensity on a scale of 0 to 10, with 0 (no pain) and 10 (worst pain).

### Measurement of functional disability

Functional disability will be assessed using the English version of the reduced Western Ontario and McMaster Universities Osteoarthritis Index (WOMAC). It consists of two dimensions: pain (five items) and function (seven items). The reliability and validity of the English version of the reduced WOMAC function scale have been established. There are two versions available one has a visual analogue response scale and the other a Likert five-point response scale [39, 40]. In the present study, a Likert five-point response scale will be used.

### Measurement of mobility by Timed Up and Go (TUG) test

The TUG test will be administered by one examiner in a quiet area [41]. A firm chair with arms (seat height of 46 cm) will be placed at one end and an object will be placed at the other end at a distance of 3-m. The test will begin with each participant sitting, back against the chair, arms on the lap, and feet just behind the starting-markings on the floor. The participants will be instructed as follows: “On the word ‘go’, stand up, walk comfortably and safely to the object at the end on the floor, walk around the object, come back, and sit all the way back in your chair.” Timing will start on the word “go” and ended when the participant returns to the chair, with back resting against the chair. A practice trial will be performed first and then followed by 2 recorded trials. The average of the 2 recorded trials will be used for data analysis.

### Adverse effects of the intervention

Adverse effects are defined as any increase in knee pain intensity perceived by the participants due to training protocol lasting for 2 days or more, or the participant requiring medications or consultation with the Physician. In addition, the risk of fall or injury to other body parts during training sessions will be considered as adverse effects. A senior Physiotherapist will supervise the entire training program.

### Sample size

The statistical software Statmate version 2 (GraphPad Software, Inc., CA, USA) was used to calculate the sample size using the primary outcome variables, the NRS scores for pain and the reduced WOMAC index for function, with a power of 80 % and a significance level of 0.05. The unpublished pilot data on the effects of retro-walking on these variables was used to calculate SD’s. We assume a clinically important difference between two intervention groups of 1.08 in NRS score (*SD* = 1.19) and 4.47 points in functional score assessed using the reduced WOMAC index (*SD* = 4.9). The sample size calculation resulted in 20 participants in each group, or 60 total participants. To allow for a potential follow-up loss of 15 %, we will recruit a minimum of 69 participants.

### Randomization, allocation concealment, and blinding

The participants who fulfil the inclusion criteria will be randomly assigned to one of three groups (A, B, and C). Blank folders will be numbered from 1 to 69 and will be given concealed codes for the group assignment by an independent researcher and kept in a safe locker. When a participant is eligible and give their consent to participate, an independent therapist will draw the next folder from the file, which will determine the group assignment. A trained research assistant blinded to group assignment will record all measurements.

### Statistical analysis

Demographic data and baseline scores of all outcome measures will be presented to evaluate baseline comparability of treatment groups. Descriptive data will be reported for each group as the mean change in the outcome measures at baseline and at the end of the trial. Data normality will be tested. Paired t-tests and one-way ANOVA with Bonferroni adjustment for multiple comparisons will be applied to compare within and between group differences of all outcomes (interval/ratio data). The Wilcoxon signed-rank test and Friedman test will be used to compare within and between group differences of all outcome measures (nominal/ordinal data). The probability level for all tests will be set at 0.05 to indicate significance. The intention-to-treat principles will be used for analysis. For missing data, the last observation carried forward method will be used. All data will be analyzed using SPSS software version 22 (SPSS, Chicago, Illinois).

## Discussion

Recently, a Cochrane review reported the effects of land-based therapeutic exercise to reduce knee pain, improve physical function and quality of life in subjects with knee OA [42]. The effectiveness of walking programs on subjects with knee OA is not well known. In a previous study, Toda [29] reported worsening of symptoms following a walking program in subjects with knee OA. However, the participants in this study were obese women with knee OA. Therefore, an increased intensity of pain in these participants may be due to the weight bearing nature of walking. In contrast, another study reported improved postural stability following long-term weight training and aerobic walking programs in subjects with knee OA [26]. In addition, Messier et al. [27] reported reduced pain and disability after combined weight training and walking program in subjects with knee OA. Furthermore, Evcik et al. [28] reported the effectiveness of home exercise and walking program (forward-walking) in the management of subjects with knee OA.

Another study reported improved knee function following conventional treatment and retro-walking program in individuals with knee OA [25]. The results of the present study will enhance our understanding on the therapeutic effects of walking (retro- and forward-walking) in subjects with knee OA, and help determine whether retro- or forward-walking or both are efficacious in the management of subjects with knee OA. Thus, our study will assess both retro- and forward-walking program in subjects with knee OA. Previous studies were limited to pain and disability outcome measures; however, in the present study, we will assess quadriceps muscle strength and mobility, in addition to pain and disability, in subjects with knee OA [25, 28].

There will be some potential limitations to the present study. The present study will not assess a long-term follow up due to poor history of patients follow up in the current hospital setting. In addition, the age group of the participants will be restricted to 40–70 years. A previous study reported impaired cognitive function and severe pain in patients with knee OA, who were 70 years or older [43]. Furthermore, the authors reported an association between worse cognitive function with poor physical function in older adults with knee OA [43]. Another study reported increased risk of falls in older adults with knee OA [44].

### Consent to publish

Not applicable.

### Ethics and consent to participate

Ethical approval has been obtained from the institutional review board (IRB), Rehabilitation Research Chair, King Saud University (file ID: RRC-2014-010). In accordance with IRB guidelines of the institution, written informed consent will be obtained from each individual who agrees to participate.

## Abbreviations

BMI: body mass index
CKC: closed kinetic chain
NRS: numerical rating scale
OA: osteoarthritis
OKC: open kinetic chain
RCT: Randomized controlled trial
TUG: timed up and go test.
WOMAC: Western Ontario and McMaster Universities osteoarthritis index
